# Gastrointestinal malignant neoplasms disguised as pneumatosis cystoids intestinalis: A case report and literature review

**DOI:** 10.1097/MD.0000000000009410

**Published:** 2017-12-22

**Authors:** Tingting Liu, Shaoheng Zhang, Hua Mao

**Affiliations:** Department of Gastroenterology, Zhujiang Hospital, Southern Medical University, Guangzhou, Guangdong, China.

**Keywords:** gastrointestinal tumor, literature review, mantle cell lymphoma, pneumatosis cystoids intestinalis

## Abstract

**Rationale:**

Pneumatosis cystoids intestinalis (PCI) is a rare disease in which gas develops in the mucosa or submucosa of the digestive tract. The etiology and pathogenesis of this disease, at present, remain unclear, and gastrointestinal malignant neoplasms may be a potentially important cause. Herein, we report a case of mantle cell lymphoma presenting as PCI as well as present a literature review of cases of suspect PCI that was definitively diagnosed as gastrointestinal neoplasms. In doing so, we highlighted cases of neoplastic pathogenesis that present as PCI.

**Patient concerns:**

A 55-year-old man was referred to our gastrointestinal department with complaints of intermittent abdominal pain, distention, diarrhea, and occasional melena that persisted for 2 months. He has a history of nasopharyngeal carcinoma.

**Diagnoses:**

Intensive, translucent, grape-like cystoids of the whole colon and small intestine were disguised as PCI upon colonoscopy and capsule endoscopy.

**Interventions:**

Right hemicolectomy and ileocecectomy were performed for intussusception and to confirm the diagnosis. Final pathology indicated that the mass was mantle cell lymphoma.

**Outcomes:**

After surgery and subsequent chemotherapy, the patient showed good recovery and no abnormal lesions were detected on colonoscopy.

**Lessons:**

As shown through this case and a literature review of similar cases of apparent PCI that was definitively diagnosed as gastrointestinal neoplasm, gastrointestinal malignant neoplasms might rarely present as PCI and neoplastic etiologies should also be considered once PCI is detected. Because most patients with malignant PCIs might inevitably experience severe complications, abdominal surgery should be considered and applied timely after unsuccessful resolution by conservative medical therapies and symptomatic treatments.

## 1 Introduction

Pneumatosis cystoids intestinalis (PCI) is a rare disease in which gas develops in the mucosa or submucosa of the digestive tract. It always presents as a benign disease and is recommended to be treated conservatively. Herein, we report a case of mantle cell lymphoma presenting as PCI as well as present a literature review of cases of suspect PCI that was definitively diagnosed as gastrointestinal neoplasms. In doing so, we highlighted the cases of neoplastic pathogenesis that present as PCI.

## 2 Case presentation

**Figure 1:**
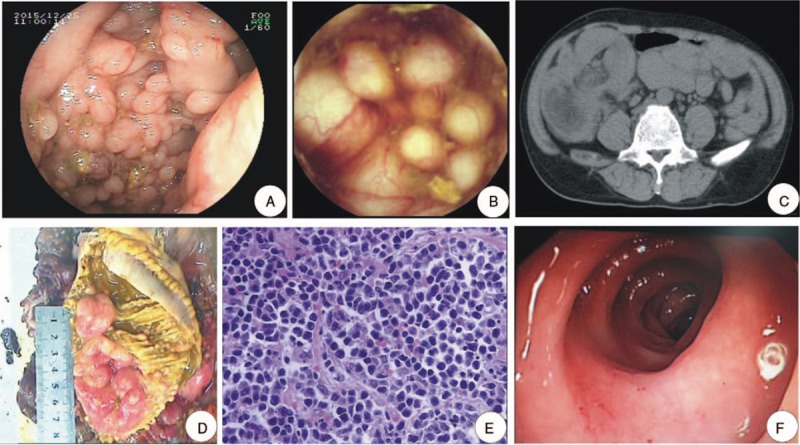
Imaging, endoscopic, and pathology information of the patient. Both colonoscopy (A) and capsule endoscopy (B) have shown intensive, translucent, grape-like cystoids with normal or congestive overlying mucosa in the lumen. When touched with biopsy forceps, these cystoids were soft or flexible, but collapsed with no fluid flowing out after mucosa biopsy. (C) An abdominal CT scan found an intussusception of the terminal ileum within the ascending colon. (D) The resected ileocecal mass is approximately 15 × 20 cm. Within the resected ileocecal segment, severe hyperemia, edema, and intensive cystoids were visible. (E) Cluster of differentiation 5-negative mantle cell lymphoma is confirmed by histologic examination (hematoxylin-eosin stain; ×40). (F) After surgery and chemotherapy, the patient showed good recovery. No abnormal lesions were detected on colonoscopy in August 2016. CD = cluster of differentiation; CT = computed tomography.

The Ethics Committee of Zhujiang Hospital approved this case report. The patient presented in this case report gave his written informed consent authorizing use and disclosure of his protected health information. A 55-year-old man was referred to our department on December 24, 2015. He complained of intermittent abdominal pain, distention, diarrhea (approximately 4–5 times/d), and occasional melena over the preceding 2 months. The patient had a history of nasopharyngeal carcinoma and was treated with radiotherapy and chemotherapy. Magnetic resonance imaging was conducted quarterly to exclude the recurrence of carcinoma. Upon physical examination, he presented with an anemic appearance and mild epigastric pain. Laboratory investigations showed an extremely low hemoglobin level (69 g/L) and positive fecal occult blood testing, whereas liver and kidney function, coagulation function, myocardial enzyme levels, serum amylase and lipase concentrations, and thyroid function were within the normal range. Electrocardiography and chest radiography findings were normal; however, abdominal ultrasound showed enlarged lymph nodes. On the second day, gastrointestinal endoscopy was conducted. During which, esophagogastroscopy indicated the presence of ulcers (A2 stage) in the gastric angle with a negative rapid urease test, whereas colonoscopy showed intensive, translucent, grape-like cystoids (Fig. 1A). The apical, adjacent mucosa of some of the cystoids appeared to be blush and erosion. When touched with biopsy forceps, these cystoids were soft or flexible, but collapsed with no fluid flowing out after mucosa biopsy. Such cystoids were also detected in the middle and the lower segments of the small intestine (Fig. 1B). With these endoscopic characteristics, endoscopists suggested PCI as the primary diagnosis; hence, venous nutrition support, mucosa protection, and symptomatic treatment were provided to the patient. However, on the fourth day, the patient experienced deteriorating abdominal pain, diarrhea, and bloody stools. Physical examination revealed a movable, tender mass in the right lower abdomen. Abdominal computed tomography revealed an intussusception of the terminal ileum within the ascending colon (Fig. 1C). Emergency exploratory laparotomy was recommended by surgeons, and right hemicolectomy and ileocecectomy were performed. A large ileocecal mass (15 × 20 cm) was detected and identified as intussusception (Fig. 1D). Within the resected ileocecal segment, severe hyperemia, edema, and intensive cystoids were visible. Surprisingly, histologic examination showed cluster of differentiation 5-negative mantle cell lymphoma (Fig. 1E). Subsequent chemotherapy, including modified-Hyper-cyclophosphamide, vincristine, doxorubicin, and dexamethasone alternating with high-dose methotrexate and cytarabine) + rituximab, high-dose cytarabine, and R-maxi-rituximab, cyclophosphamide, doxorubicin, vincristine, prednisone alternating with rituximab were administrated successively since January 13, 2016. As the patient recovered well, and there were no abnormalities detected by colonoscopy (Fig. 1F) and full gastrointestinal contrast, stoma closure was conducted on August 16, 2016.

## 3 Literature review

As gastrointestinal neoplasms might exhibit a similar appearance as PCI, we performed a literature search to further address PCI and its potential neoplastic pathogenesis. Five databases, including PubMed, Web of science, Embase, WanFang Data, and China National Knowledge Infrastructure, were searched up to July 2017 by using the following search query: (pneumatosis cystoids) AND (gastri∗ OR intestin∗ OR jejunum OR jejun∗ OR ileum OR ileo∗ OR cecum OR cecal OR colon OR sigmoid OR rectum) AND (cancer OR tumor OR malignant OR neoplasms OR lymphoma). No language limit was applied and non-English articles were translated when necessary. Eventually, 18 reports, including 19 cases, were identified (Table 1).^[1–18]^ Of these cases, 10 were men and 9 were women, and their average age was 64.3 ± 15.3 years. The most common symptom was abdominal pain (7/19, 36.8%), followed by diarrhea (4/19, 21%), distention (5/19, 26.3%), bloody stool (4/19, 21%), nausea (4/19, 21%), vomiting (5/19, 26.3%), hypodynamia (3/19, 15.7%), loss of weight (5/19, 26.3%), fever (4/19, 21%), and jaundice (2/19, 10.5%). These symptoms appeared to be nonspecific. The determined malignant neoplasms included cardiac cancer (n = 1), duodenal carcinoma (n = 2), lower gastrointestinal carcinoma (n = 8, 1 in jejunum, 1 in ileocecum, 3 in colon, 1 in rectum, and 2 in sigmoid), duodenal metastasis from cholangiocellular carcinoma (n = 2), lymphoma (n = 5), and gastrointestinal sarcoidosis (n = 1). Remarkably, these aforementioned malignant neoplasms all led to the complication of gastrointestinal stenosis, which ultimately required surgical removal and subsequent histologic examination.

**Table 1:**
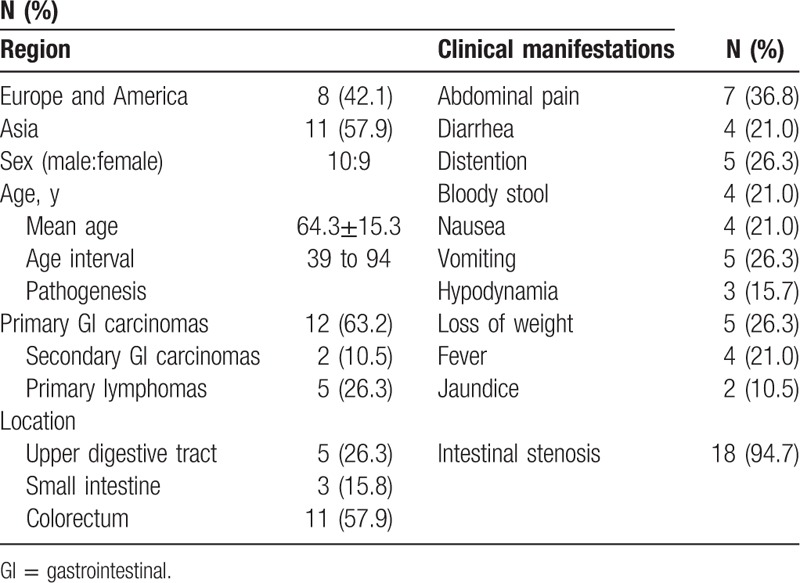
Clinical information of the selected cases (N = 19).

## 4 Discussion

PCI is an uncommon disease with an unknown etiology that is characterized by the presence of gas within the submucosa or subserosa of the intestine. Although some cases and case series have been reported, the vast majority of these reports refer to sporadic cases and have not yet identified a general incidence. However, systematic analysis might provide some general characteristics of PCI. In a retrospective review of PCI, Koss^[19]^ found a 3.5:1 male-to-female ratio for the occurrence of PCI in patients aged 30–50 years, and Jamart^[20]^ observed a 3:1 male-to-female ratio (41–50 years of age) for PCI. In this literature review, we observed a 10:9 male-to-female ratio and an average age of 64.3 ± 15.3 years. Moreover, the most common localization of gas was in the submucosa (69.9%), with PCI occurring more commonly in the colon.^[21]^ Morris et al^[22]^ indicated that the incidence of PCI was 46% in the colon, 27% in the small intestine, and only 7% in both the colon and small intestine. Meanwhile, Wu et al ^[21]^ recorded the approximate trend of location (colon vs. small bowel, 1.3:1) in a Chinese cohort.

Though first described by Du Vernoy ^[23]^ in autopsy specimens in 1730 and subsequently named by Mayer as PCI in 1825, PCI is still poorly understood. Currently, there are several major theories to explain the abnormal accumulation of gas. For example, “the mechanical theory” means that intestinal obstruction, inflammatory bowel disease, ischemic bowel disease, gastroenteric tumor, anorectal surgery, and bowel preparation or colonoscopy resulting in intestinal wall injury or increased intraluminal pressure serve as the causative agent in PCI of intramural gas.^[24,25]^ Meanwhile, the bacterial theory refers to the production of gas by gas-forming bacteria that enters the mucosal barrier through mucosal rents or increased mucosal permeability. Indirect support for this theory was obtained by the successful treatment of PCI with antibiotics. Other theories, including the pulmonary theory and the chemical theory or nutritional deficiency theory have also been described.^[16]^ Although many theories can account for the etiology or pathogenesis of PCI, no theory is sufficient to account for the entire pathologic processes. Similarly, the mechanisms of disease in the 19 patients described in this study are unclear. Based upon our experiences and observations, the mechanical theory and the bacterial theory best support our findings.

The clinical manifestations of PCI are varied. According to our literature review, the common manifestations include abdominal pain, diarrhea, distention, bloody stool, loss of weight, fever, and some other nonspecific symptoms. Such manifestations highlight the similarities between the benign group and the malignant group. When the cysts increase in size and become larger, the cysts may commonly cause obstruction by internal or external compression of the bowel lumen. Statistics from Wu et al ^[21]^ showed us a complication incidence of 16.3% (39/239), which mainly includes intestinal obstruction (51.3%, 20/39) or intestinal perforation (35.9%, 14/39). Notably, there are relatively fewer cases reported as intussusception associated with PCI. In 2016, Itazaki et al ^[26]^ performed a literature review and found 9 reports of such cases. For this reason, interpretation or analysis of the pathogenesis of intussusception associated with PCI is needed.

PCI may be found by laparotomy, radiology, or endoscopic examinations, and can distinguish between benign or malignant tumors through pathological examination. Once confirmed, its management might be challenging for clinicians. Currently, there is no consensus on the appropriate management. Conservative medical therapies and symptomatic treatments are recommended empirically, whereas surgical treatment should be considered when severe complications, such as bowel obstruction, intussusception, and massive hemorrhage, occur. In our literature review, all patients with PCI derived from gastrointestinal malignant tumors ultimately required surgical removal, thereby indicating that clinicians must be mindful of such potential complications and the deteriorations of PCI. Abdominal surgery should be considered and performed at the most appropriate time if possible.

## 5 Conclusion

Gastrointestinal malignant neoplasms can present as PCI, and neoplastic etiologies should also be considered once PCI is detected. As most patients with malignant PCIs may inevitably experience severe complications, abdominal surgery should be considered and applied emergently after unsuccessful resolution by conservative medical therapies and symptomatic treatments.

## Acknowledgments

The authors would like to thank the Department of Gastroenterology, Zhujiang Hospital, Southern Medical University for supporting this work.

## References

[1] Mohd Rosli R Raju D Luck A B-cell lymphoma presenting as pneumatosis intestinalis: a case report. ANZ J Surg 2014;84:892–3. 25348917 10.1111/ans.12750

[2] Rahim H Khan M Hudgins J Gastrointestinal sarcoidosis associated with pneumatosis cystoides intestinalis. World J Gastroenterol 2013;19:1135–9. 23467442 10.3748/wjg.v19.i7.1135 PMC3582003

[3] Fong KY Siao FY Yen HH Cecal pneumatosis intestinalis in obstructing sigmoid cancer: emergency metallic stenting. Am J Emerg Med 2014;32:395.e1–3. 10.1016/j.ajem.2013.10.040 24275044

[4] Bilici A Karadag B Doventas A Gastric pneumatosis intestinalis associated with malignancy: an unusual case report. World J Gastroenterol 2009;15:758–60. 19222105 10.3748/wjg.15.758 PMC2653449

[5] Soon MS Yen HH Soon A Endoscopic ultrasonographic appearance of gastric emphysema. World J Gastroenterol 2005;11:1719–21. 15786559 10.3748/wjg.v11.i11.1719 PMC4305963

[6] Schorner W Schworer I [Rare causes of multiple filling defects of the colon. Report on a colonic involvement in non-Hodgkin's lymphoma and pneumatosis cystoides coli]. Rofo 1982;136:719–20. 6213503 10.1055/s-2008-1056137

[7] Phothong N Swangsri J Akaraviputh T Colonic stenting for malignant colonic obstruction with pneumatosis intestinalis: a case report. Int J Surg Case Rep 2016;26:38–41. 27448227 10.1016/j.ijscr.2016.07.012 PMC4957606

[8] Seto T Koide N Taniuchi N Pneumatosis cystoides intestinalis complicating carcinoma of the small intestine. Am J Surg 2001;182:287–8. 11587694 10.1016/s0002-9610(01)00710-3

[9] O’Connell DJ Thompson AJ Pneumatosis coli in non-Hodgkins lymphoma. Br J Radiol 1978;51:203–5. 630189 10.1259/0007-1285-51-603-203

[10] Stuart M Pneumatosis coli complicating carcinoma of the colon. Report of a case. Dis Colon Rectum 1984;27:257–9. 6714034 10.1007/BF02553800

[11] Chou YH Hsu HL Lee JC Emphysematous colitis of ascending colon with portal venous air caused by diffuse large B-cell lymphoma. J Clin Oncol 2010;28:e496–7. 20679597 10.1200/JCO.2010.29.1229

[12] Holt RW Dekker J Gastric pneumatosis intestinalis associated with cholangiocarcinoma. South Med J 1986;79:79–80. 2418509 10.1097/00007611-198601000-00025

[13] Shomali W Davis MP Gastric pneumatosis: an unexpected complication of intractable vomiting in gastrointestinal cancers. J Pain Symptom Manage 2015;49:e3–4. 25623921 10.1016/j.jpainsymman.2014.12.006

[14] Yan JH Chang Y Gao J A case of cardiac cancer complicated with esophageal pneumatosis cystoides. China Journal of Endoscopy 2004;10:3–13. (Article in Chinese).

[15] Liu H San HX Lu F Pneumatosis coli secondary to the colon carcinoma: a case report. Journal of North Pharmacy 2011;08:87–8.

[16] Fang HB Zhao B Ma QZ Pneumatosis cystoids intestinalis of ascending colonic. Report of a case. The Journal of Practical Medicine 2006;22:1748.

[17] Wang SY Chen XM A case of pneumatosis coli in canceration with villous tubular adenoma of the colon and literature review. Chinese Journal of Digestive Endoscopy 2009;26:272–3. (Article in Chinese).

[18] Lin H Ma LL Li SD Clinical analysis of 2 cases of pneumatosis coli. Zhejiang Clinical Medical Journal 2006;8:1204 (Article in Chinese).

[19] Koss LG Abdominal gas cysts (pneumatosis cystoides intestinorum hominis); an analysis with a report of a case and a critical review of the literature. AMA Arch Pathol 1952;53:523–49. 14923068

[20] Jamart J Pneumatosis cystoides intestinalis. A statistical study of 919 cases. Acta Hepatogastroenterol (Stuttg) 1979;26:419–22. 525221

[21] Wu LL Yang YS Dou Y A systematic analysis of pneumatosis cystoids intestinalis. World J Gastroenterol 2013;19:4973–8. 23946603 10.3748/wjg.v19.i30.4973 PMC3740428

[22] Morris MS Gee AC Cho SD Management and outcome of pneumatosis intestinalis. Am J Surg 2008;195:679–82. 18424288 10.1016/j.amjsurg.2008.01.011

[23] Du Vernoi GJ. Aer intestinorum tam subextima guam intima tunica inclusus. Vol 51730.

[24] Nancy Fu YT Kim E Bressler B Pneumatosis intestinalis after colonoscopy in a Crohn's disease patient with mucosal healing. Inflamm Bowel Dis 2013;19:E7–8. 22147522 10.1002/ibd.22840

[25] Wertkin MG Wetchler BB Waye JD Pneumatosis coli associated with sigmoid volvulus and colonoscopy. Am J Gastroenterol 1976;65:209–14. 937319

[26] Itazaki Y Tsujimoto H Ito N Pneumatosis intestinalis with obstructing intussusception: a case report and literature review. World J Gastrointest Surg 2016;8:173–8. 26981192 10.4240/wjgs.v8.i2.173 PMC4770172

